# Effect of Polymer Removal on the Morphology and Phase of the Nanoparticles in All-Inorganic Heterostructures Synthesized via Two-Step Polymer Infiltration

**DOI:** 10.3390/molecules26030679

**Published:** 2021-01-28

**Authors:** Diana Berman, Yuchen Sha, Elena V. Shevchenko

**Affiliations:** 1 Materials Science and Engineering Department and Advanced Materials and Manufacturing Processes Institute, University of North Texas, 1155 Union Circle, Denton, TX 76203, USA; 2 Center for Nanoscale Materials, Argonne National Laboratory, 9700 S. Cass Ave, Argonne, IL 60439, USA; ysha@anl.gov; 3 Institute of Advanced Studies (IAS), College of Chemistry and Molecular Sciences, Wuhan University, Wuhan 430072, China

**Keywords:** block copolymers, sequential infiltration synthesis, magnetic nanoparticles, ceramic composites

## Abstract

Polymer templates play an essential role in the robust infiltration-based synthesis of functional multicomponent heterostructures with controlled structure, porosity, and composition. Such heterostructures are be used as hybrid organic–inorganic composites or as all-inorganic systems once the polymer templates are removed. Using iron oxide/alumina heterostructures formed by two-step infiltration of polystyrene-block-polyvinyl pyridine block copolymer with iron and aluminum precursors from the solution and vapor-phases, respectively, we show that the phase and morphology of iron oxide nanoparticles dramatically depend on the approach used to remove the polymer. We demonstrate that thermal and plasma oxidative treatments result in iron oxide nanoparticles with either solid or hollow morphologies, respectively, that lead to different magnetic properties of the resulting materials. Our study extends the boundaries of structure manipulations in multicomponent heterostructures synthesized using polymer infiltration synthesis, and hence their properties.

## 1. Introduction

Polymer templates have been widely used for the design of a broad range of functional composite materials and all-inorganic structures [1,2,3,4]. The combination of polymer properties, such as the ability to form conformal coatings [5], response to external stimuli [6,7], and variability in their chemistry [8,9,10,11] enable precise grafting of hybrid materials synthesized by polymer infiltration with inorganic precursors. The resulting heterostructures are used as organic–inorganic hybrids [12,13] or as all-inorganic heterostructures formed once the polymer template is removed [14,15]. Infiltration of the polymer can be performed from vapor [11], solution [15], or consequently from both phases [15,16] enabling synthesis of unary and multicomponent structures.

The polymer template after the infiltration can be removed by various postprocessing approaches [17,18,19,20]. Among them, thermal annealing and ultraviolet ozone treatment are the most common techniques used for polymer removal in structures synthesized by sequential infiltration synthesis (SIS). While polymer removal approaches utilize a wide range of processing conditions, the focus of the previous studies was on the infiltration processes rather than on the polymer removal effect [14,15]. The selection of the polymer removal technique is usually made considering mainly acceptability of the residual carbon and stability of the substrate that was used for the growth of conformal coating or structure by SIS [21]. It was shown that thermal annealing in oxygen-containing flow, in addition to the fast polymer removal, can lead to the densification of the porous structures and better mechanical stability [22,23]. In turn, UV ozone treatment is slower and more gentle for substrates but may lead to the trapping of residual carbon [16,24,25]. Previous studies on alumina synthesized by SIS and based on the infiltration of polymer block copolymers (BCPs) with trimethylaluminum (TMA) did not reveal a difference in alumina structures obtained by UV ozone treatment and thermal annealing polymer removal [26]. However, our recent study on the synthesis of porous all-inorganic PdO_x_/alumina nanoheterostructures indicated that the technique used for polymer template removal drastically affects the size of the PdO_x_ nanoparticles (NPs) uniformly distributed in the alumina matrix [16].

Here we investigate the effect of the polymer removal regime on the morphology and composition of the multicomponent inorganic heterostructures synthesized by two-step infiltration from solution and vapor-phases using iron oxide/alumina heterostructures as a model system. Iron oxides can be represented by different polymorphs [27], oxidation states of iron, and various morphologies including spheres, rings, wires, disks, rods, and hollow structures [28,29,30,31,32,33,34]. The polymorphic nature and different morphologies of iron oxide make it a promising candidate to explore the effect of the polymer removal process performed in the synthesis of all-inorganic structures.

We report that the two-step infiltration approach enables the synthesis of iron oxide nanoparticles (NPs) in the alumina matrix with their structure and composition being significantly affected by the polymer removal process. Iron oxide/alumina heterostructures were formed using the approach previously utilized to synthesize PdO_x_ NPs uniformly distributed in the alumina matrix [16]. The approach involves infiltration of the functional material precursor via swelling-based infiltration (SBI) step in solution followed by vapor-phase infiltration of the alumina matrix precursor via SIS [16,35,36,37]. We demonstrate that thermal and plasma oxidative treatments result in iron oxide nanoparticles with either solid or hollow morphologies with different magnetic properties.

## 2. Results and Discussion

**Figure 1:**
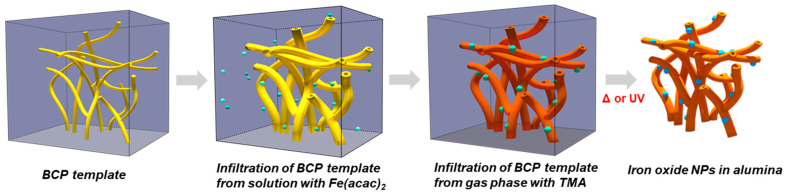
Schematic of synthesis of iron oxide nanoparticles (NPs) in a porous alumina matrix using a two-step infiltration process. The polar domains of polymer block copolymers (BCP) (here PS-P4VP) are infiltrated first with the iron precursor (Fe(acac)_2_) from ethanol solution followed by the infiltration with vapors of alumina precursors (TMA and H_2_O). Note that the immersion of BCP into ethanol solution is accompanied by its swelling. The BCP template is removed by either thermal annealing or plasma treatment resulting in the formation of all-inorganic heterostructure consisting of iron oxide NPs in porous alumina.

The samples of iron oxide NPs in the alumina matrix were synthesized using a two-step infiltration process that includes: (i) infiltration of PS/P4VP BCP polymer template with an iron precursor such as iron acetylacetonate or Fe(acac)_2_ (FeAA) in ethanol solution and (ii) SIS infiltration of alumina from the vapor-phase precursor such as TMA (Figure 1). The iron oxide NPs in alumina samples were obtained as a result of the polymer template removal process using thermal annealing at 450 °C in an oxygen atmosphere or UV ozone treatment. Previously, this approach has been used to synthesize palladium, ruthenium, and cobalt oxide NPs in a porous alumina matrix [16]. In our earlier study, we noticed that the polymer removal process influenced the size of NPs. Here we chose the system such as iron oxide that can present in different crystalline and oxidation states, as well as NP morphologies. We aim to explore to what extent the polymer removal step affects the structure, size, and morphology of NPs in all-inorganic heterostructures synthesized via a two-step infiltration approach. For this, we tested two common processes of polymer removal, UV ozone treatment, and thermal annealing.

**Figure 2:**
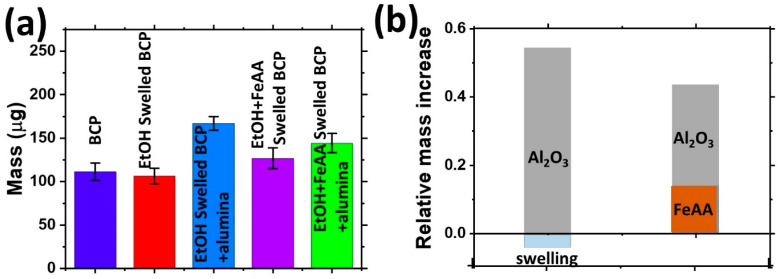
Quartz crystal microbalance (QCM) monitoring of the swelling and infiltration processes of PS-P4VP template from the Fe(acac)_2_ solution and from vapor-phase: (**a**) the mass increase during different stages of polymer modifications, and (**b**) relative mass increase upon material infiltration as compared to the initial mass of the polymer film during the vapor-phase infiltration (left bar) and during solution and vapor-phase infiltration (right bar).

The infiltration processes with iron precursor from solution and with aluminum precursor from gas phase were characterized using the quartz crystal microbalance (QCM) technique (Figure 2). Swelling of the BCP in pure ethanol by immersing the QCM into the beaker with 50 mL of ethanol at 75 °C for 1 h produces a small mass decrease associated with, probably, partial dissolution of the BCP during the micelle opening (relative mass change as compared to initial polymer film mass is −3%). Analysis of the ethanol after the swelling indicated the presence of a small fraction of the dissolved polymer [38]. Exposure of the BCP (~110 μg) to the SBI process when the swelling is performed in ethanol with 0.5 wt% of Fe(acac)_2_ leads to a mass increase (~10 μg) attributed to the absorption of iron oxide precursor inside the polar domains of the BCP (relative mass change is 14%). The following exposure of the samples to the SIS infiltration of alumina leads to a 58% relative mass increase (~64 μg) in the case of the BCP after swelling in ethanol and a 28% relative mass increase (~30 μg) in the case of the BCP after swelling in ethanol with Fe(acac)_2_. The difference in the infiltration capacity of the polymers is attributed to the different molar weights of the inorganic precursors as well as to the conversion of the Fe(acac)_2_ precursor to iron oxide upon exposure to water vapors during the SIS process.

**Figure 3:**
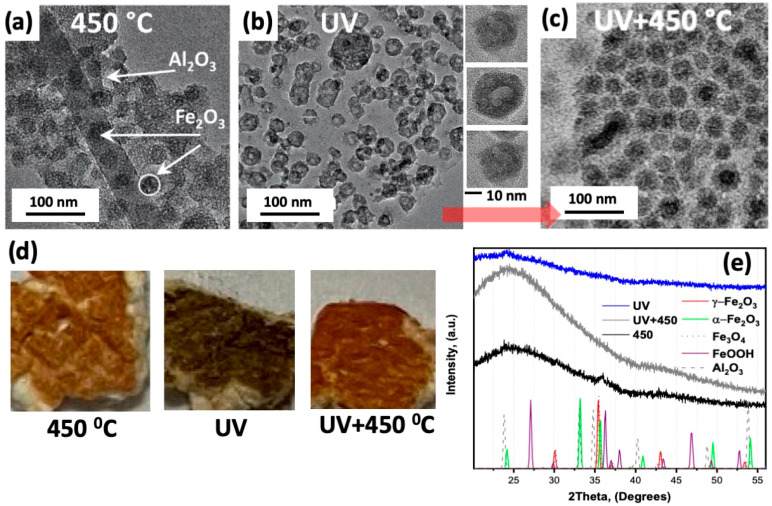
TEM images of the samples after the polymer removal using (**a**) the thermal annealing process at 450 °C, (**b**) UV ozone treatment (the images on the right demonstrate higher resolution TEM images of the representative individual NPs), and (**c**) UV ozone treatment followed by oxidative annealing at 450 °C. (**d**) Photographs and (**e**) XRD data of iron oxide/alumina heterostructures obtained by different polymer removal strategies. The samples shown in (**d**) were deposited on fiberglass for better contrast.

After polymer removal, the obtained heterostructures were analyzed by TEM (Figure 3). For that, the fragments of the samples were dispersed in acetone and deposited on the TEM grid. Our results revealed that both UV ozone treatment and the thermal annealing process led to the formation of NPs with a diameter of 20–30 nm with higher contrast that are embedded in the amorphous alumina matrix. It is reasonable to assume that these NPs correspond to the oxidized form of iron. Interestingly, the sample received after thermal annealing at 450° is brownish and the TEM image shows solid nanoparticles (Figure 3a,d). In the case of the UV ozone treatment, the resulting heterostructure has a dark grey color (Figure 3) that could indicate the presence of unoxidized iron. Also, the NPs look like hollow structures [34,39]. These results suggest that oxidative thermal annealing and UV ozone treatment lead to the formation of different oxidized forms of iron. Previously, we observed a more reduced nature of palladium in the PdO_x_/alumina samples synthesized using a similar combined solution/vapor infiltration process even after a rather aggressive oxidative treatment at 450 °C [16]. The reduced nature of Pd we attributed to the reduction of the Pd(2+) cations adsorbed in polar domains of the BCP by TMA. Here we also suggest that TMA can reduce adsorbed Fe(3+) cations. Their further exposure to the H_2_O initiates the formation of oxidized forms of iron, most likely oxides or hydroxides. The XRD analysis indicates the highly amorphous nature of the formed nanostructures (Figure 3e). However, when the polymer template was removed using UV ozone plasma the XRD spectrum has a low-intensity peak at 2θ = 24.1° that agrees with (1 0 −2) peak position of α-Fe_2_O_3_ (Figure 3e). In turn, the XRD spectrum of the sample annealed at 450 °C has a broad low-intensity peak centered at 2θ = 35.8° (Figure 3e) that can correspond to γ-Fe_2_O_3_, Fe_3_O_4_, α-Fe_2_O_3,_ and FeOOH. The broad peak at smaller angles covers the peaks corresponding to α-Fe_2_O_3_ and FeOOH. Interestingly, oxidative annealing of the UV ozone plasma-treated sample transforms the dark grey color to brown and the NPs change their morphology from hollow to spherical of larger diameter (Figure 3c,d). Such a transformation could be explained by the Kirkendall effect [40,41] assuming that the initial hollow structures contain not fully oxidized iron (e.g., FeO and/or Fe_3_O_4_ or hydroxides) and their inner part is accessible for oxidation. Oxidation of the inner part results in the expansion of the oxide and the formation of NPs of spherical morphology. The analysis of the TEM images reveals that some NPs have several voids and that the voids could be of different sizes (Figure 3b, right panel). These observations are in agreement with the Kirkendall effect. The Kirkendall effect has been previously reported to result in the formation of hollow iron oxide NPs upon oxidation of iron NPs [33]. The other shape of NPs that is possible based on the analysis of the TEM images is a donut. However, in this case, we should also observe the NPs with a rectangular shape (the side projection of “donut-like” NPs), that have not been found in the TEM images (Figure 3b). Thus, we can conclude that the hollow shape of iron oxide NPs is similar to previously reported hollow structures [40,41]. Upon thermal annealing, the XRD spectrum of the UV ozone-treated sample becomes similar to that of heterostructures directly annealed at 450 °C (Figure 3e). The size of the formed NPs is larger than that of the initial hollow NPs by ~20%. The densities of γ-Fe_2_O_3_ and FeOOH are ~4.9 g/cm^3^ and ~4.25 g/cm^3^, respectively. The densities of most common forms of reduced iron such as Fe_3_O_4_, FeO, and Fe are ~5.17, ~5.74 and 7.874 g/cm^3^, respectively. The density difference between oxidized and less oxidized forms also suggests that it is reasonable to expect the expansion of the volume of NPs containing reduced iron upon oxidation.

**Figure 4:**
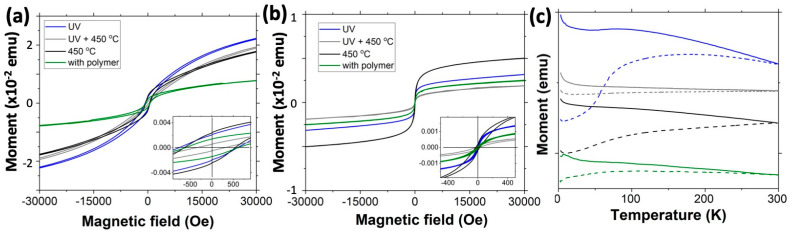
Magnetization (M) hysteresis loops at 5 K and 300 K (**a**,**b**, respectively), and (**c**) zero-field cooled (ZFC, dashed curves) and field cooled (FC, solid curves) curves obtained with 100 Oe applied magnetic field for PS-P4VP template infiltrated with FeAA and alumina, and the iron oxide/alumina heterostructures with the polymer removed by UV ozone treatment and by thermal annealing.

Magnetic property measurements (Figure 4) indicate that all samples demonstrated significantly lower magnetization, M_s_, (less than ~20 emu/g calculated by dividing the magnetization by the mass of iron oxide NPs in the samples) at 5 K as compared to bulk ferromagnetic γ-Fe_2_O_3_ (74 emu/g) and ferrimagnetic Fe_3_O_4_ (84 emu/g) [42]; however, these were significantly higher than those of antiferromagnetic hematite α-Fe_2_O_3_ (0.4 emu/g) [43] and goethite FeOOH (0.31 emu/g) [44] (Figure 4a). The low saturation magnetization can be a result of a surface spin disorder in ferro or ferrimagnetic NPs due to broken spins and canting of the surface spins; thus, the saturation requires a very high magnetic field. It is worth noting that low saturation magnetization (less than 30 emu/g) has been previously reported for polycrystalline hollow iron oxide nanoparticles [42], which is significantly lower as compared to solid NPs [45]. However, lower magnetization can also indicate the contribution of ferro/ferri- and antiferromagnetic phases. An interesting observation is that even the polymer template infiltrated with FeAA revealed magnetic properties (Figure 4a) showing that magnetic nanostructures were formed prior to oxidative polymer removal. The highly amorphous nature of the magnetic structures enables their soft magnetic behavior. However, here we observed coercivities of ~ 600 Oe at 5K for all samples including the as-infiltrated sample, annealed at 450 °C, and UV ozone-treated samples. Such coercivities are likely due to the interfacial effects [42,46]. After reaching nearly saturation magnetization, the surface spins remain pinned at 5 K (Figure 4a). Thermal annealing at 450 °C of the UV ozone-treated sample results in a decrease of magnetization and coercivity (Figure 4). The decrease of magnetization agrees with further oxidation of samples. Fe and Fe_3_O_4_ have a saturation magnetization of ~222 emu/g and 84 emu/g [42], respectively, which is higher than the saturation magnetization of γ-Fe_2_O_3_ with 74 emu/g and antiferromagnetic hematite or goethite. While the rather amorphous nature of the samples does not allow establishing the direct structure–magnetic properties’ correlations, the magnetic measurements data in combination with TEM and XRD data indicate the structural transformation of the magnetic NPs. The decrease of the coercivity of the UV ozone-treated samples after thermal annealing can be attributed to the transformation of hollow NPs into spherical, and hence lowering the concentration of the interfacial defects. At 5 K, the UV ozone plasma-treated sample initially has lower magnetization as compared to the sample annealed at 450 °C, which increases rapidly and exceeds the magnetization of the annealed sample (Figure 4a). However, at 300 K, the sample annealed at 450 °C has a higher magnetization over the whole applied magnetic field range (Figure 4b). Also, at 300 K, the sample with polymer and the sample that underwent UV ozone treatment show zero coercivities, while the sample after the thermal annealing shows coercivity of ~35 Oe, which could be due to structural defects [47] or indicative of some contribution of antiferromagnetic phase(s) that is (are) characterized by high coercivities [43,44]. The zero field cooling (ZFC) and field cooling (FC) data indicate the ferromagnetic response of all samples (Figure 4c). Magnetic measurement data demonstrate that the polymer removal strategy has a significant effect on the magnetic properties of the hybrid iron oxide/alumina heterostructures.

## 3. Materials and Methods

### 3.1. Polymer Template Preparation

The samples of iron oxide incorporated in the amorphous alumina matrix were prepared using the previously reported two-step infiltration process [16] via the swelling-based infiltration (SBI) followed by a vapor-phase sequential infiltration synthesis (SIS). Polymer templates for the infiltration were prepared using a 3 wt% toluene solution of polystyrene-block-polyvinyl pyridine (PS-b-P4VP) polymer with 75k-b-25k g/mol molar weight spin-coated on the quartz crystal microbalance (QCM) substrates or infiltrated in a support matrix of the paper filter (Sigma Aldrich, Inc., MO, USA). The resulting samples were dried on a hot plate at 100 °C in nitrogen flow for one hour to remove any residual solvent.

### 3.2. Infiltration from Swelling Solution

SBI of the BCP templates was performed using a solution of the iron oxide precursor to synthesize iron oxide NPs. For this, a solution of the iron acetylacetonate, Fe(acac)_2_ (FeAA), in 50 mL of ethanol at 0.5% concentration optimized in our previous studies [16] was used to initiate SBI of the materials inside the polar P4VP domains during their selective swelling in ethanol at 75 °C for 1 h. Identical polymer templates were swelled in ethanol without FeAA to characterize the loading of the materials during the infiltration steps. After SBI, the samples were dried at room temperature overnight in nitrogen glow to remove any residual ethanol from the BCP and to stabilize the swelled and infiltrated structure of the BCP. As was shown in our earlier studies [21], swelling in ethanol results in the formation of additional porous channels that significantly increase the infiltration depth of the BCP.

### 3.3. Vapor-Phase Infiltration

Alumina infiltration of the prepared BCP with FeAA composites was performed using the SIS process in an atomic layer deposition system (Cambridge ALD system). The samples were exposed to 5 cycles of trimethylaluminum (TMA) and water vapors at 90 °C [21,38]: 10 mTorr of the TMA precursor was introduced with 20 sccm nitrogen flow into the reactor for 400 s; the excess of the reactant was evacuated for 10 s followed by introducing of 10 mTorr of H_2_O for 120 s; the chamber was then purged with 100 sccm of nitrogen to remove not-infiltrated byproducts. The process allows the synthesis of amorphous alumina matrix [5,26] that is used here to provide the support for iron oxide NPs.

### 3.4. Polymer Removal

The polymer template after the infiltration was removed by 3-hour thermal annealing at 450 °C in oxygen flow (20 sccm) using a ThermoFisher tube furnace or by 24-hour room temperature treatment in the UV ozone cleaner (UVOCST16 × 16 OES, 254 nm UV wavelength).

### 3.5. QCM Analysis

The efficiency of the infiltration was analyzed with the Quartz Crystal Microbalance technique [35,36,37,48] previously used for precise monitoring of surface modifications upon exposure to different liquids and thermal treatment processes. In short, changes in the resonant frequency of oscillations of AT-cut QCM crystals (with initial resonant frequency ~ 5 MHz) with ~200 nm thick uniform BCP films spin-coated on the titanium electrodes were monitored during different stages of the BCP exposure to swelling, SBI, and SIS. The QCMs fixed in a Teflon holder were analyzed using the SRS QCM200 system. Observed changes in the resonant frequency of the oscillations were correlated with the changes in the mass of the polymer films to estimate the amount of the infiltration material. For this, the negative change in frequency $\left(\delta f_{film}\right)$ of the QCM upon mass adsorption can be calculated by:

(1) $$\delta f_{film}=-\frac{2f^{2}}{A\sqrt{\rho_{q}\mu_{q}}}\Delta m$$

where *f* is the fundamental frequency of the QCM, Δ*m* is mass change, *A* is the surface area of the QCM, *ρ_q_* = 2648 kg/m^3^ and *µ_q_* = 2.947 × 10^6^ kg·m/s^2^ are the density and the shear modulus of quartz, respectively.

### 3.6. Magnetic Properties

Magnetic properties of the resulting iron oxide in alumina samples were characterized with a DynaCool-14 (Quantum Design, San Diego, CA, USA) Physical Properties Measurement System (PPMS) machine using a vibrating sample magnetometer (VSM) option. The samples (~20 µg in mass) synthesized from BCP templates supported by the paper filter matrix were placed in gelatin capsules and fixed to the quartz rod. Zero field cooling (ZFC) and field cooling (FC) measurements were performed at 100 Oe upon heating (after initial cooling at a zero magnetic field) and cooling the samples, respectively.

### 3.7. Characterization

Transmission electron microscopy (TEM) analysis of the iron oxide in alumina matrix samples was performed using a JEOL 2100F microscope (JEOL Ltd., Tokyo, Japan) on the samples crushed and deposited on the carbon mesh TEM grid. The XRD data were measured using the Bruker D2 X-ray diffractometer with Cu Kα source of irradiation. The data were fitted using an inorganic crystal structure database [49].

## 4. Conclusions

Our study provides insights into the effect of polymer removal on the hybrid structures obtained via a two-step infiltration process by combining swelling-based infiltration (SBI) of iron oxide and sequential infiltration synthesis (SIS) of aluminum oxide. We show that iron oxide NPs uniformly distributed in a porous alumina matrix can be obtained by infiltration of PS-P4VP with FeAA from ethanol solution followed by infiltration with TMA from the vapor-phase. We demonstrate that solid spherical iron oxide NPs of similar size are formed in a porous alumina matrix by thermal treatment under airflow at 450 °C, while hollow iron oxide NPs are formed as a result of UV ozone treatment. Interestingly, such hollow structures transform into spherical NPs upon subsequent thermal annealing. Regardless of polymer removal strategy, iron oxide NPs are rather amorphous. The magnetic measurement data highlight the impact of the polymer removal strategy, indicating the significant difference in saturation magnetizations and coercivities of the samples obtained using UV ozone treatment and thermal annealing. Our study extends the boundaries of structure manipulations in multicomponent heterostructures synthesized using polymer infiltration synthesis, and hence their properties.

## Data Availability

The data presented in this study are available on request from the corresponding author.

## Sample Availability

Samples of the compounds are available from the authors.
